# Prevalence and associated factors of illicit drug use among university students in the association of southeast Asian nations (ASEAN)

**DOI:** 10.1186/s13011-017-0096-3

**Published:** 2017-04-06

**Authors:** Siyan Yi, Karl Peltzer, Supa Pengpid, Indri Hapsari Susilowati

**Affiliations:** 1 KHANA Center for Population Health Research, No. 33, Street 71, Tonle Bassac, Chamkar Mon, Phnom Penh Cambodia; 2 Center for Global Health Research, Touro University California, Vallejo, USA; 3 ASEAN Institute for Health Development, Mahidol University, Salaya, Thailand; 4 Department of Research & Innovation, University of Limpopo, Turfloop, South Africa; 5 HIV/AIDS/STIs/and TB (HAST), Human Sciences Research Council, Pretoria, South Africa; 6 Faculty of Public Health, Universitas Indonesia, Depok, Indonesia

**Keywords:** Illicit drug use, Prevalence, Substance use, Risk factors, University students, ASEAN

## Abstract

**Background:**

Illicit drug use among university students has been recognized as a global public health issue in recent years. It may lead to poor academic performance that in turn leads to poor productivity in their later life. This study explores prevalence of and factors associated with illicit drug use among university students in the Association of Southeast Asian Nations (ASEAN).

**Methods:**

This multi-country cross-sectional study was conducted in 2015 in Cambodia, Indonesia, Laos, Malaysia, Myanmar, the Philippines, Singapore, Thailand and Vietnam. A multi-stage cluster sampling was used to select undergraduate students from one or two universities in each country for self-administered questionnaire survey. Multivariate logistic regression analyses was performed to explore risk factors related to illicit drug use.

**Results:**

Participants included 7,923 students with a mean age of 20.6 years (SD = 2.8), ranging from 18–30 years. The overall prevalence of frequent (≥10 times), infrequent (1–9 times) and ever (at least once) illicit drug use in the past 12 months was 2.2, 14.7, and 16.9%, respectively. After adjustment, male students were significantly less likely to be infrequent (1–9 times vs. never), but significantly more likely to be ever users compared to females. Compared to those living with parents/guardians, students living away from parents/guardians were significantly less likely to be frequent (≥10 times vs. never) and infrequent users. Students from lower-middle-income countries were significantly more likely to be frequent and infrequent users, but significantly less likely to be ever users compared to those from upper-middle or high-income countries. Students with poor subjective health status were significantly more likely to be frequent users compared to those who reported good subjective health status. Students who reported binge drinking in the past month were significantly more likely to be infrequent users, but significantly less likely to be ever users.

**Conclusions:**

Our findings indicate that prevalence of illicit drug use among university students in the ASEAN region varied by country. Concerted social intervention programs should be designed to address related health and behavioral problems such as illicit drug use and alcohol drinking with particular emphasis on at-risk subgroups of this young population.

## Background

According to the World Health Organization (WHO), risk behavior is defined as “a specific form of behavior, which is proven to be associated with increased succeptability to a specific disease or ill-health” [1]. Risk taking encompasses behaviors that at the same time involves the chance of a beneficial outcome as well as possible negative or harmful consequences [2, 3]. Risk-taking behaviors increase during adolescence and young adulthood [4, 5]. These behaviors are associated with heightened reactivity to emotions and a still immature ability to self-regulate [6, 7], making adolescence and youth a period of high vulnerability to the negative consequences of risk-taking [8]. Several studies on risk behaviors among these young populations have highlighted its long-term consequences on both physical and psychological health of the youngsters [9–11].

College years is a period characterized by transition, intense academic pressures as well as independence and separation from parental supervision [12]. During this period, opportunities to experiment with psychoactive substances, including illicit drugs, increases [13]. Illicit drug use among university students has been recognized as a global public health issue [14] and globally evident in recent years [12, 15–17]. It may lead to poor academic performance that in turn leads to poor productivity in their later life [15, 18].

Recently, several epidemiological studies have been conducted to estimate the prevalence of illicit drug use in university students in different countries. In the United States, the past-year prevalence of illicit drug use ranged from 11 to 17%, and the prevalence of current use ranged from 6 to 8% [14]. Peltzer and Pengpid, using data collected in eight countries in Africa and three countries in the Caribean, reported that the prevalence of infrequent (1–9 times) and frequent (≥10 times) illicit drug use in the past 12 months was 17 and 4%, respectively [19]. In the United Kingdom, 5% of the study sample from seven universities reported regularly using illicit drugs, and 25% reported occasional use of them [20]. Only a few studies have been conducted in Asian countries, and information on types of illicit drugs included in the studies were limited. In a study in India, 7% of the students reported cannabis use [16]. In the Middle East, lifetime prevalence of illicit drug use was 14% in Kuwait [15], and the prevalence of current use was 8% in Iran [17].

Several risk factors associated with illicit drug use among university students have been identified in different countries. These factors included socio-demographic characteristics such as male gender [17, 20, 21], high family income [15, 22], living on or off campus on their own, living in an upper-middle- or high-income country [19] and financial burden [20]. Illicit drug use is also associated with the use of other substances including tobacco smoking and alcohol drinking [17, 19, 20, 23], internal states such as anxiety and depression [24] and violent behavior such as physical fight [19, 25]. Identified protective factors include older age [15, 19], higher level of religiosity [19, 26] and living with parents [17].

Only a few studies on risk and protective factors for illicit drug use have been conducted among university students in the ASEAN region. In most of the existing studies, illicit drug use is combined with other substances in the analyses [16, 18], making the interpretation of the findings difficult. Therefore, this study was conducted to explore the prevalence of and factors associated with illicit drug use among university students in Cambodia, Indonesia, Laos, Malaysia, Myanmar, the Philippines, Singapore, Thailand and Vietnam.

## Methods

### Participants and sampling

This multi-country cross-sectional study was conducted between 2013 to 2015 as part of a larger investigation of a range of health behaviors in undergraduate university students by a network of researchers in the participating countries (see Acknowledgments).

A multi-stage cluster sampling method was used to select 400 male and 400 female students aged 16–30 years by trained research assistants in the respective countries. First, a convenient sampling method was used to select one or two universities in each country, considering administration and logistic limitation. In each university, undergraduate students were surveyed in classrooms selected through a stratified random sampling procedure. A university department formed a cluster and was used as a primary sampling unit. One department was randomly selected from each faculty. For each selected department, undergraduate courses offered by the department were randomly ordered. All students attending the selected class were eligible to participate; there were no exclusion criteria. We included no incentive for participation, and there were no penalties for refusing to complete the survey. Participation rates in most of the participating countries were more than 90%, except for Indonesia (86%) and Myanmar (73%).

### Data collection training

A two-day training was conducted for all data enumerators and supervisors on the study protocol, data collection method and research ethics. The training focused on familiarity with the survey materials, interview techniques, privacy assurance and confidentiality. Quality control strategies, such as rechecking and reviewing the questionnaires after administration, and resolving issues that might arise during the fieldwork were also emphasized. The data collection supervisors were instructed to perform regular reviews with the data enumerators to monitor progress and settle any issues occurring during the process.

### Questionnaire development

A structured self-administered questionnaire was developed in English, and then translated into the national language (Bahasa, Khmer, Myanmar, Lao, Thai, Vietnamese) of the participating countries. Another bilingual translator, who had no knowledge of the original instrument, back-translated the re-conciliated target language version. The translated questionnaire was pretested with a sample of students to ensure that the wording and contents were culturally suitable and clearly understandable.

Illicit drug use was assessed with a question, “In the past 12 months, how often have you taken any drugs other than those prescribed by health care providers?” Response options ranged from 1 = 0 time to 4 = 10 or more times.

Self-rated health status was assessed by a single item, “In general, would you say that your health is: Excellent, Very good, Good, Fair or Poor?” [27].

Ten items of the Center for Epidemiologic Studies Depression Scale (CES-D) was used to assess depressive symptoms, and an individual with a score of ≥15 would be classified as having severe depressive symptoms [28] (Cronbach’s alpha = 0.73).

For other substance use, a yes/no question, “Are you currently using one or more of tobacco products (cigarettes, snuff, chewing tobacco, cigars, etc.)?” [29] was used to assess current tobacco use. Past month binge drinking was assessed with one item of the Alcohol Use Disorder Identification Test: “In the past month, how often did you have five or more (for men) and four or more (for women) alcoholic drinks on one occasion?” Response options included 0 = never, 1 = less than once a month, 2 = Once a month, 3 = Once a week and 4 = Every day or almost every day” [30].

Physical fight was measured using a question, “During the past 12 months, how many times were you in a physical fight?” Response options ranged from ‘0 times’ to ‘12 or more times’ [31].

Academic performance was assessed with one question, “How would you rate your academic performance?” The response option ranged from 1 = excellent to 5 = poor. Socio-demographic characteristics included age, gender, residential status and subjective socioeconomic background. Family economic background was assessed by rating their family background as wealthy (within the highest 25% in terms of wealth), quite well off (within the 50–75% range), not very well off (within the 25–50% range) or quite poor (within the lowest 25%). Responses were collapsed into two groups, being poor or not well off and wealthy or quite well off [27].

### Data analyses

Data analyses were performed using STATA software version 11.0 (Stata Corporation, College Station, Texas, USA). The prevalence of the illicit drug use and characteristics of participants were calculated as a percentage (%). Multivariate logistic regression analyses were undertaken separately for frequent illicit drug use (ten or more times), infrequent illicit drug use (1–9 times) and ever use in the past 12 months. All variables were simultaneously included in the model.

## Results

### Sample characteristics

The total sample included 7,923 undergraduate students, of whom 62.5% were female with a mean age of 20.6 years (SD = 2.8, range of 18–30 years). The sample size ranged from 639 in Myanmar to 1,132 in Cambodia. Overall, 2.2 and 14.7% of the respondents reported frequent and infrequent illicit drug use in the past 12 months, respectively. As shown in Table 1, there was a country variation in the prevalence of both infrequent (ranging from 0.2% in Cambodia to 45.7% in Laos) and frequent (ranging from 0.0% in Cambodia to 5.5% in Laos) illicit drug use.

**Table 1:** Prevalence of different levels of illicit drug use among university students in nine countries in the ASEAN

Country	Sample	Illicit drug use in the past 12 months		
	Number	Never use (0 times)	Infrequent use (1–9 times)	Frequent use (ten or more times)
Cambodia^a^	1134	1132 (99.8)	2 (0.2)	0 (0.0)
Indonesia^a^	981	532 (54.2)	397 (40.5)	52 (5.3)
Laos^a^	806	394 (48.9)	368 (45.7)	44 (5.5)
Malaysia^b^	1023	974 (95.2)	47 (4.6)	2 (0.2)
Myanmar^a^	639	545 (85.3)	78 (12.2)	16 (2.5)
Philippines^a^	757	634 (83.8)	99 (13.1)	24 (3.2)
Singapore^c^	887	781 (88.0)	87 (9.8)	19 (2.1)
Thailand^b^	884	784 (88.7)	83 (9.4)	17 (1.9)
Vietnam^a^	817	808 (98.9)	3 (0.4)	6 (0.7)
All	7923	6584 (83.1)	1164 (14.7)	175 (2.2)

*Abbreviations*: *ASEAN* Association of Southeast Asian Nations

^a^Lower-middle income country; ^b^Upper-middle income country; ^c^High-income country [36]

### Factors associated with illicit drug use

Table 2 shows results of multivariate analyses exploring factors associated with illicit drug use in the past 12 months. After controlling for other covariates simultaneously included in the model, male students were significantly less likely to be infrequent users (1–9 times vs. never) (AOR = 0.71, 95% CI = 0.53–0.96), but significantly more likely to be ever users (AOR = 1.34, 95% CI = 1.02–1.76) compared to female students. Compared to those living with their parents or guardians, students living away from parents were significantly less likely to be frequent (≥10 times vs. never) (AOR = 0.60, 95% CI = 0.39–0.91) and infrequent (AOR = 0.76, 95% CI = 0.66–0.88) users. Regarding country income categories, students from lower-middle-income countries were significantly more likely to be frequent (AOR = 3.36, 95% CI = 1.28–8.82) and infrequent (AOR = 2.83, 95% CI = 2.41–3.33), but significantly less likely to be ever users (OAR = 0.25, 95% CI = 0.10–0.61) in the past 12 months compared to those from upper-middle or high-income countries. Students with poor subjective health status were significantly more likely to be frequent users compared to those who reported good subjective health status (AOR = 1.84, 95% CI = 1.26–2.68). In relation to other substance use, students who reported binge drinking in the past month were significantly more likely to be infrequent illicit drug users (AOR = 1.92, 95% CI = 1.03 = 3.57), but significantly less likely to be ever users (AOR = 0.52, 95% CI = 0.30–0.90).

**Table 2:** Factors associated with illicit drug use in the past 12 months among university students in nine countries in ASEAN

Variables in the model	Frequent use (≥10 times vs. never)	Infrequent use (1–9 times vs. never)	Never vs. ever use
	AOR (95% CI)^a^	AOR (95% CI)^b^	AOR (95% CI)^c^
Age (in years)			
18–19 (31.4%)	Reference	Reference	Reference
20–21 (41.5%)	1.29 (0.65–2.57)	1.19 (0.70–2.01)	0.83 (0.48–1.43)
22–30 (27.0%)	0.88 (0.42–1.82)	1.33 (0.65–2.75)	0.79 (0.39–1.59)
Gender			
Female (62.5%)	Reference	Reference	Reference
Male (37.5%)	1.03 (0.68–1.55)	0.71 (0.53–0.96)^*^	1.34 (1.02–1.76)^*^
Family background			
Quite well off/wealthy (36.0%)	Reference	Reference	Reference
Quite poor, not well off (64.0%)	1.13 (0.80–1.60)	0.81 (0.54–1.23)	1.18 (0.80–1.73)
Living situation^d^			
With parents (35.7%)	Reference	Reference	Reference
Away from parents (64.3%)	0.60 (0.39–0.91)^*^	0.76 (0.66–0.88)^***^	1.36 (0.97–1.92)
Country income categories			
Upper-middle/high income (38.7%)	Reference	Reference	Reference
Lower middle income (61.3%)	3.36 (1.28–8.82)^*^	2.83 (2.41–3.33)^***^	0.25 (0.10–0.61)^**^
Poor subjective health status (4.7%)	1.84 (1.26–2.68)^**^	0.99 (0.59–1.67)	0.91 (0.58–1.44)
Depression (severe) (9.2%)	1.13 (0.75–1.71)	1.26 (0.86–1.86)	0.80 (0.58–1.10)
Current tobacco use (4.0%)	3.35 (0.88–12.71)	2.09 (0.76–5.72)	0.42 (0.14–1.23)
Binge drinking (past month) (6.1%)	1.67 (0.66–4.27)	1.92 (1.03–3.57)^*^	0.52 (0.30–0.90)^*^
Having been in physical fight in the past 12 months (6.9%)^d^	1.39 (0.66–2.95)	1.25 (0.92–1.69)	0.77 (0.55–1.09)
Poor academic performance (11.2%)	0.50 (0.11–2.40)	0.34 (0.10–1.13)	2.79 (0.82–9.44)

*Abbreviations*: *AOR* adjusted odds ratio, *ASEAN* Association of Southeast Asian Nations, *CI* confidence interval

^a^Hosmer & Lemeshow Chi-square = 6.16, *P* = 0.630; Nagelkerke R^2^ = 0.11

^b^Hosmer & Lemeshow Chi-square = 42.79, *P* < 0.001; Nagelkerke R^2^ = 0.15

^c^Hosmer & Lemeshow Chi-square = 31.37, *P* < 0.001; Nagelkerke R^2^ = 0.15

^d^Data from Cambodia were excluded

^***^ *P* < 0.001; ^**^ *P* < 0.01; ^*^ *P* < 0.05

## Discussion

To the best of our knowledge, this is the first multi-country study on illicit drug use among university students in the ASEAN region, in which standardized tools were employed. We found a wide range of the prevalence of illicit drug use ranging from as low as 0.2% in Cambodia to as high as 46% in Laos for infrequent use and from 0.0% in Cambodia to 5.5% in Laos for frequent use in the past 12 months. Overall, the prevalence of illicit drug use among university students across the nine ASEAN countries is considerably high and comparable with the prevalence rates reported in other countries [15, 17, 20, 22]. This finding supports the global concern that illicit drug use in university students is a significant public health issue that requires particular attention [32].

Several socio-demographic and behavioral characteristics have been found to be associated with different levels of illicit drug use. Living with parents or guardians and living in a lower-middle-income country increased the risk of both frequent and infrequent illicit drug use. The relationship between living with parents or guardians and the increased odds of illicit drug use was also found in a previous study [17] but not in another one [19]. While high-family income was reported to be a risk factor for illicit drug use in Kuwait [15] and Brazil [22], the relationship was not found among university students in our study. Our finding of the increased risk of illicit drug use among students living in a lower-income country has added an important information to the literature in this research area. This finding could be explained by the geographical location of these countries that is close to major drug production zones [14].

Interestingly, the results in this study suggest that living with parents seems to have no significant protectice effects on illicit drug use among university students in the ASEAN region. After controlling for other potential confounders, living away from parents or guardians decreased the risk of both frequent and infrequent illicit drug use among students in this study. A recent study of university students in the Northern Ireland, Wales and England found that illit drug use was less common among students living with their parents [20]. Similarly, living on campus increased the likelihood of initiating marijuana use among American students during the freshman year [33]. Another study in Iran also reported that living away from parent home was a risk factor for lifetime illicit drug use [17].

This study did not find an association between levels of academic performance and illicit drug use, which has been reported in previous studies [15, 18, 20]. Illicit drug use was associated with poor academic performance among university students in Kuwait [15] and Pakistan [18]. In contrast, a study among university students in the United Kingdom reported that illicit drug use was more common among students who reported that their academic performace was better than that of their peers [20]. The non-significant relationship between illicit drug use and academic performance in our study is possibly explained by the low rates of illicit drug use in some countries, and the onset of the use was fairly recent. In this situation, the negative effects of academic performance may have not yet developed.

Male students in this study were significantly more likely to engage in both infrequent and lifetime illicit drug use compared to females. Higher prevalence rates were also found in previous studies [17, 20, 21], but the gender difference was not statistically significant. In this study, infrequent illicit drug use was found to be more common in female than in male students. It is possible that female students were more likely to use unprescribed medications than male students. Due to the nature of the data, we were not able to distinguish the unprescribed medications from illicit drug use. This will need further investigation, as to what particular drugs had been used.

Consistent with findings from other studies, we found that both frequent and infrequent illicit drug use was significantly associated with the use of other substances, including binge drinking. Similar findings have been reported in several previous studies [17, 20, 23, 25] and may indicate that certain problem behaviors, in particular different types of substance use, cluster together. Illicit drug use and other substance abuse such as binge drinking may be a clustering risk that may occur and become a culture during youth and young adulthood [34]. Using one drug may lower the barriers of taking another drug [33]. According to the European Monitoring Centre for Drugs and Drug Addiction, alcohol intoxication may lead to ill-considered decisions about consuming illicit drugs, thereby biasing the individual towards engaging in polydrug use [35]. Because the same university students are at risk for different substance use, an integrated approach for preventing substance use among these young population may be warranted and necessary.

Several limitations of this study should be noted. First, beause of the cross-sectional nature of the data, no causal relationships could be derived. Second, this study included only students from one or two universities in each country, therefore the findings may not be generalized to university students at a national or regional level. Third, the self-reported measures employed in this study may result in recall and reporting biases that are potential for both over-reporting and under-reporting due to social desirability. Forth, in the measurement of illicit drug use, the types of the drugs were not specified. This limited our understanding of the common form and related risk factors that might be different in the participating countries. Finally, most of the measures were modified from other previous studies, and have not been validated in the individual context of study settings. Notwithstanding these malfeasances, the findings of this study offer first and foremost implications for policy development and future research the ASEAN region.

## Conclusions

Illicit drug use among university students in the ASEAN region varied by country, and the overall prevalence is comparable to the rates reported in other parts of the world. Particular attention is needed for some countries in the region including Laos, Indonesia and the Philippines, where the prevalence rates are particularly high. Our findings indicate that synergistic collaboration needs to be built between the ASEAN member states to respond to this important issue. Concerted social intervention programs should be designed to address related health and behavioral problems such as illicit drug use and binge drinking with particular emphasis on subgroups of this young at-risk population.

## Abbreviations

AOR: Adjusted odds ratio
ASEAN: Association of Southeast Asian Nations
CES-D: Centres for Epidemiologic Studies Depression Scale
CI: Confidence interval
SD: Standard deviation

## References

[1] World Health Organization (WHO) (1998). Health promotion glossary.

[2] Arnett JJ (2000). Emerging adulthood. A theory of development from the late teens through the twenties. Am Psychol.

[3] Boyer TW (2006). The development of risk-taking: a multi-perspective review. Dev Rev.

[4] Ernst M, Pine DS, Hardin M (2006). Triadic model of the neurobiology of motivated behavior in adolescence. Psychol Med.

[5] Gardner M, Steinberg L (2005). Peer influence on risk taking, risk preference, and risky decision making in adolescence and adulthood: an experimental study. Dev Psychol.

[6] Steinberg L (2008). A social neuroscience perspective on adolescent risk-taking. Dev Rev.

[7] Steinberg L (2010). A dual systems model of adolescent risk-taking. Dev Psychobiol.

[8] Steinberg L (2005). Cognitive and affective development in adolescence. Trends Cogn Sci.

[9] Mohammadpoorasl A, Ghahramanloo AA, Allahverdipour H (2013). Risk-taking behaviors and subgrouping of college students: a latent class analysis. Am J Mens Health.

[10] Gonzalez J, Ward BW (1994). Adolescents’ perceptions of their risk-taking behavior. Adolescence.

[11] Resnick MD, Bearman PS, Blum RW, Bauman KE, Harris KM, Jones J (1997). Protecting adolescents from harm. Findings from the national longitudinal study on adolescent health. JAMA.

[12] Sommet A, Ferrières N, Jaoul V, Cadieux L, Soulat JM, Lapeyre-Mestre M (2012). Use of drugs, tobacco, alcohol and illicit substances in a french student population. Therapie.

[13] Locke GW, Shilkret R, Everett JE, Petry NM (2015). Interpersonal guilt and substance use in college students. Subst Abus.

[14] Degenhardt L, Hall W (2012). Extent of illicit drug use and dependence, and their contribution to the global burden of disease. Lancet.

[15] Bajwa HZ, Al-Turki AS, Dawas AM, Behbehani MQ, Al-Mutairi AM, Al-Mahmoud S (2013). Prevalence and factors associated with the use of illicit substances among male university students in Kuwait. Med Princ Pract.

[16] Gupta S, Sarpal SS, Kumar D, Kaur T, Arora S (2013). Prevalence, pattern and familial effects of substance use among the male college students -a north Indian study. J Clin Diagn Res.

[17] Mohammadpoorasl A, Ghahramanloo AA, Allahverdipour H, Augner C (2014). Substance abuse in relation to religiosity and familial support in Iranian college students. Asian J Psychiatr.

[18] Khattak MA, Iqbal N, Khattak SR, Ullah I (2012). Influence of drugs on student performance: a qualitative study in Pakistan university students. Interdis J Contem Res Bus.

[19] Peltzer K, Pengpid S (2016). Correlates of illicit drug use among university students in Africa and the Caribbean. J Psychol Afr.

[20] El Ansari W, Vallentin-Holbech L, Stock C (2015). Predictors of illicit drug/s use among university students in northern Ireland, wales and England. Glob J Health Sci.

[21] Osman T, Victor C, Abdulmoneim A, Mohammed H, Abdalla F (2016). Epidemiology of substance use among university students in Sudan. J Addict.

[22] Silva LV, Malbergier A, Stempliuk Vde A, de Andrade AG (2006). Factors associated with drug and alcohol use among university students. Rev Saude Publica.

[23] Ayvasik HB, Sümer HC (2010). Individual differences as predictors of illicit drug use among Turkish college students. J Psychol.

[24] Cho SB, Llaneza DC, Adkins AE, Cooke M, Kendler KS, Clark SL (2015). Patterns of substance use across the first year of college and associated risk factors. Front Psychiatry.

[25] Dukarm CP, Byrd RS, Auinger P, Weitzman M (1996). Illicit substance use, gender, and the risk of violent behavior among adolescents. Arch Pediatr Adolesc Med.

[26] Gomes FC, de Andrade AG, Izbicki R, Moreira Almeida A, Oliveira LG (2013). Religion as a protective factor against drug use among Brazilian university students: a national survey. Rev Bras Psiquiatr.

[27] Wardle J, Steptoe A (1991). The european health and behaviour survey: rationale, methods and initial results from the united kingdom. Soc Sci Med.

[28] Andresen EM, Malmgren JA, Carter WB, Patrick DL (1994). Screening for depression in well older adults: evaluation of a short form of the CES-D (center for epidemiologic studies depression scale). Am J Prev Med.

[29] World Health Organization (WHO) (1998). Guidelines for controlling and monitoring the tobacco epidemic.

[30] Babor TF, Higgins-Biddle JC, Saunders JB, Monteiro M (2001). AUDIT: the alcohol use disorder identification test.

[31] Centers for Disease Control and Prevention (CDC). Global School-based Student Health Survey (GSHS). Retrieved on 23 July 2015 from http://www.cdc.gov/gshs/. Accessed 23 July 2015.

[32] Dennhardt AA, Murphy JG (2013). Prevention and treatment of college student drug use: A review of the literature. Addict Behav.

[33] Suerken CK, Reboussin BA, Sutfin EL, Wagoner KG, Spangler J, Wolfson M (2014). Prevalence of marijuana use at college entry and risk factors for initiation during freshman year. Addict Behav.

[34] Newbury-Birch D, Walshaw D, Kamali F (2001). Drink and drugs: from medical students to doctors. Drug Alcohol Depend.

[35] European Monitoring Centre for Druds and Drug Addiction (2009). Polydrug use: patterns and responses.

[36] World Bank. New Country Classifications. 2016. Retrieved at http://blogs.worldbank.org/opendata/new-country-classifications-2016. Accessed 20 Jul 2016.

